# Characteristics Associated with Acute-Phase Response following First Zoledronic Acid Infusion in Brazilian Population with Osteoporosis

**DOI:** 10.1155/2021/9492883

**Published:** 2021-12-29

**Authors:** Bruno S. A. Ferreira, Bernardo M. da Cunha, Luciana P. Valadares, Larissa A. Moreira, Frederico G. A. Batista, Cristiane da F. Hottz, Marina M. P. Lins, Gabriel G. R. Magalhães, Luanna M. de Arruda, Sergio H. R. Ramalho

**Affiliations:** ^1^Rheumatology Private Office, Teresópolis 25963-081, Brazil; ^2^Department of Internal Medicine-Rheumatology, Sarah Network of Rehabilitation Hospitals, Brasilia 70335-901, Brazil; ^3^Department of Internal Medicine-Endocrinology, Sarah Network of Rehabilitation Hospitals, Brasilia 70335-901, Brazil; ^4^Department of Internal Medicine-Nutrology, Sarah Network of Rehabilitation Hospitals, Brasilia 70335-901, Brazil; ^5^Department of Internal Medicine-Gerontology, Sarah Network of Rehabilitation Hospitals, Brasilia 70335-901, Brazil; ^6^Department of Internal Medicine-Palliative Care, Sarah Network of Rehabilitation Hospitals, Brasilia 70335-901, Brazil; ^7^Department of Clinical Research, Coordinator, Hospital Brasília DASA, Brasilia 71681-603, Brazil

## Abstract

We aimed to evaluate characteristics associated with acute-phase response (APR) following first zoledronic acid infusion in a Brazilian cohort. This retrospective cohort study enrolled all adults with osteoporosis who underwent a first zoledronic acid infusion at our centre between June 2015 and June 2019. Clinical demographics (age, sex, diabetes, smoking, body mass index, and previous oral bisphosphonate use) and laboratory data (calcium, parathyroid hormone, renal function, and serum 25-hydroxyvitamin D and carboxy-terminal crosslinked telopeptide of type 1 collagen [CTX], both before and after infusion) were compared between patients with and without APR. We evaluated association magnitude between the presence of APR and clinical variables through logistic regression. This study enrolled 400 patients (women, 80%). APR was observed in 24.5% (*n* = 98) of patients. The mean symptom duration in days was 3.5 ± 2.8. Patients with APR were younger (67 ± 12 vs. 71 ± 11 years; *p*=0.001), used oral bisphosphonates less frequently (34% × 50%; *p*=0.005), and had greater baseline CTX (0.535 ng/mL [0.375, 0.697] × 0.430 [0.249, 0.681]; *p*=0.03) and ΔCTX (−69 [−76; −50] × −54 [−72; −23]; *p*=0.002) than those without APR. The other variables were similar between the groups. Only ΔCTX was associated (OR, 0.62; 95% CI 0.41–0.98) with APR after accounting for age and bisphosphonate use. APR occurred in 24.5% of the cohort. Younger age and absence of prior oral bisphosphonate use were associated with APR following first zoledronic acid infusion. APR was associated with ΔCTX (but no other variables) after adjusting for these factors.

## 1. Introduction

Osteoporosis is a bone condition caused by a reduction in bone strength. This disease is highly prevalent worldwide and causes devastating consequences in terms of health (fragility, fractures, disability, and death) and healthcare costs [1–3]. In most cases, oral (alendronate or risedronate) or venous (zoledronic acid) bisphosphonates are the first-line therapy [2, 4–6]. This drug class has been administered to millions of patients and is implemented in the treatment of virtually all metabolic bone diseases [7].

Zoledronic acid is a venous treatment administered annually that guarantees full treatment throughout the year [8]. However, its use is linked to acute-phase reactions (APR). Consequently, treatment with zoledronic acid may decrease adherence to the next dose.

Mechanisms mediating APR require additional study. Up to now, studies have demonstrated proinflammatory cytokine (interleukin-1, interleukin-6, tumour necrosis factor alpha, and interferon gamma) elevation following bisphosphonate administration (mainly for venous bisphosphonates: pamidronate and zoledronic acid) [9–13]. These cytokines are primarily released by specific peripheral blood lymphocytes (*γδ* T cells) [10]. Nevertheless, recent studies on osteoimmunity have shown a pivotal role of the macrophage-osteoclast axis in the inflammatory response [14].

The frequency of such reactions is widely variable (13–81%) [15, 16] and seems to be influenced by race, age, vitamin D levels, smoking, diabetes, and prior use of oral bisphosphonates [17–22]. Some preemptive drugs for reducing the risk of APR were tested in previous investigations, which found that paracetamol 0.65–1.0 g administered every 6 h for 3 days following infusion demonstrated a protective effect [23, 24], in contrast to 300 mg of paracetamol administered twice daily [16]. Previous studies have likewise examined dexamethasone (4 mg daily for 3–7 days), methylprednisolone (40 mg for 2 days), and ibuprofen, which all demonstrated effectiveness in alleviating symptoms [24–26].

Observational studies in real-world scenarios have demonstrated the importance of providing evidence regarding treatment effectiveness and long-term safety outside controlled research settings [27]. However, data regarding APR in patients with osteoporosis following their first zoledronic infusion is scarce, especially in Latin America, where zoledronic infusion is administered less frequently than in other regions [15].

Hence, this study aimed to describe the frequency of APR as well as factors associated with APR in a Brazilian population with osteoporosis receiving their first zoledronic acid infusion in daily practice settings.

## 2. Materials and Methods

We conducted a real-world retrospective cohort study at the Bone Unit of a tertiary rehabilitation centre in Brasilia, Federal District, Brazil. Patients with osteoporosis who received their first zoledronic acid infusion between June 2015 and June 2019 were considered eligible. We enrolled all osteoporosis patients who visited our centre; patients were diagnosed through densitometric and/or clinical criteria [4, 28, 29]. Patients treated with zoledronic acid for other metabolic bone diseases (e.g., Paget disease, osteogenesis imperfecta, and bone fibrous dysplasia) were excluded. We abstracted clinical and demographic characteristics (age, sex, diabetes, smoking, body mass index, and previous use of oral bisphosphonates) and laboratory data (calcium, parathyroid hormone, vitamin D before and after infusion, carboxy-terminal crosslinked telopeptide of type 1 collagen [CTX] before and after infusion, and glomerular filtration rate according to the Chronic Kidney Disease Epidemiology Collaboration (CKD-EPI, creatinine equation)) from patients' electronic medical records, including preinfusion exams collected up to 6 months preceding infusion and postinfusion exams collected up to 1 year after infusion. Age was included in models and sensitivity analyses as a continuous variable and categorised according to tertiles.

Medical guidelines recommend administration of cholecalciferol to patients with osteoporosis and 25-hydroxyvitamin D deficiency [30]. The association between low 25-hydroxyvitamin D levels and APR remains controversial [18–20]. To better investigate this association, isolated serum 25-hydroxyvitamin D concentration should ideally be measured immediately before infusion; however, this is sometimes unfeasible in a real-life cohort. To better characterise the relationship between vitamin D levels and APR in our study, we collected postinfusion values in addition to baseline values. According to reference categories describing normal (≥30 ng/dL) or low (<30 ng/dL) vitamin D values [30], the subset of patients who had both (pre and post) values available were divided into two groups: (a) the low-low group, in which levels of 25-hydroxyvitamin D were low before and after infusion (assuming low values during infusion), and (b) the normal-normal group, in which both concentrations were normal (assuming normal levels during infusion). The proportions of patients in each group were compared based on the presence or absence of APR using the chi-square test. Nonetheless, given the aim of this sensitivity analysis, patients who changed 25-hydroxyvitamin D level status (i.e., low levels before infusion and normal levels after infusion, or normal levels before infusion and low levels after infusion) were not included this analysis, since we could not presume patients' 25-hydroxyvitamin D level status during the infusion.

We investigated APR following a zoledronic acid dose of 5 mg infused in a 100 mL saline solution for 20–30 min. Patients were administered with paracetamol 750 mg immediately after the infusion and instructed to take it every 6–8 h during the following 2–3 days. An acute-phase reaction was defined as fever, fatigue, malaise, diffuse pain, headache, and/or digestive symptoms starting within 28–36 h after zoledronic acid infusion [9]. The duration of symptoms was calculated as the average and median of symptom duration in days.

Continuous data analysis was performed using unpaired *t*-test and Mann-Whitney test for normally and nonnormally distributed variables, respectively. For categorical data, we used the chi-square test. All statistical test parameters were two-sided, and a *p* value of 0.05 was considered the level of statistical significance. The strength of associations between exposures and APR was tested using logistic regression models, unadjusted and adjusted for age, previous oral bisphosphonate use, baseline CTX, and the difference between baseline and postinfusion CTX (ΔCTX). Results were expressed as odds ratios (ORs) and 95% confidence intervals (CIs). Nonnormally distributed variables were log-transformed to fit regression models. Statistical analysis was performed using Stata software version 14 (StataCorp, College Station, TX, USA).

The study was approved by the Ethics Committee of Sarah Network of Rehabilitation Hospitals (Ethical Appreciation Presentation Certificate number: 32665420.9.0000.0022) and was performed in accordance with the tenets of the Declaration of Helsinki.

## 3. Results

We analysed 400 patients among 428 osteoporosis patients receiving their first zoledronic acid infusion, since 28 patients were lost to follow-up or had missing registration (regardless of whether they had a reaction or not). We compared both groups (those with and without missing data) to verify any significant differences (Table 1). The overall frequency of acute-phase responses was 24.5%, which were mostly observed in women (Table 2). The average duration of symptoms was 3.5 ± 2.8 days and the median duration was 3 days. Diabetes was infrequent (14.5%); 227 (57%) patients had never smoked. The mean CTX was 0.516 ng/mL before infusion and 0.218 ng/mL after infusion (dropping 58% from baseline). The average vitamin D level was 33.6 ng/mL. The mean eGFR was 60 mL/min.

APR and prior bisphosphonate use were significantly associated with younger age (Table 2), such that the likelihood of reaction was reduced by 3% per incremental year of age. Those who used oral bisphosphonates had a 49% reduced likelihood of reaction (Table 3). No subject in this study was on hormone replacement therapy.

CTX preceding infusion was slightly but significantly higher in patients who had APR (Table 2), although this association did not persist when accounting for age and prior oral bisphosphonate use. ΔCTX was greater in patients who had APR than in those who did not (Table 2), and this association persisted even after accounting for age and oral bisphosphonate use (Table 3).

We did not observe differences between groups for other clinical or laboratory characteristics (Table 2). Given that only 258 patients had baseline CTX values and 180 patients had ΔCTX measurements, we performed a sensitivity analysis to identify potential baseline imbalances among those without missing data (Table 4). Among this subsample of 180 patients, those with APR were significantly younger, used oral bisphosphonates less frequently, and had a higher baseline CTX and a greater ΔCTX (i.e., similar distributions were observed in the full sample). As a sensitivity approach, exclusively among women, no substantial differences were observed from the primary analysis, except for more active smoking associated with APR (Supplemental Table S1), while among men, a greater ΔCTX was the only variable associated with APR (Supplemental Table S2). Also, additionally adjusting the model to sex, the magnitudes of association persisted (Supplemental Table S3).

Serum 25-hydroxyvitamin D mean values did not differ between groups, even when analysing patients who did not change their vitamin D status.

## 4. Discussion

In this large sample real-life cohort, an acute-phase reaction was observed in 24.5% of consecutive patients with osteoporosis after their first zoledronic acid infusion. Older age and previous use of oral bisphosphonates were independently associated with lower APR frequency. Greater baseline CTX and ΔCTX levels were also associated with APR. Only the association with ΔCTX persisted when accounting for age and previous bisphosphonate use. These findings may help to identify patients with an increased likelihood of APR in the Brazilian population, which has been relatively understudied.

We observed an APR frequency lower than that previously reported (30 to 80%) [16, 18–20, 26], but this study confirms previous findings from the HORIZON study with respect to the Latin American population [17]. This may be partially explained by the routine use of prophylactic medications, given that other studies reported higher APR incidences when pharmacologic prophylaxis was precluded [16, 22]. In agreement with previous studies, we also found that age and previous oral bisphosphonate use are factors associated with acute-phase response [18, 20, 22, 24]. Interestingly, baseline CTX and particularly ΔCTX were also associated with APR, which, to our knowledge, has not been reported previously.

The association between 25-hydroxyvitamin D and APR seems to be related to baseline levels. In contrast to our findings, Reid et al. showed a relationship between 25-hydroxyvitamin D levels and APR [17]. Other researchers have shown that low levels represent a risk factor for acute-phase response [18, 20], while normal levels may be protective [21]. Additionally, 25-hydroxyvitamin D may be related to the severity of APR, rather than its presence or absence. Popp et al. reported that the mean value was significant only when non-acute-phase responders were compared to severe responders, while no difference was found compared to mild or moderate responders.

Bertoldo et al. [18] found a large difference in vitamin D levels between those with APR (25.40 ± 14.22 ng/mL) and those without APR (47.18 ± 22.95). If necessary, the study subjects were supplemented with cholecalciferol (400 IU/day) and calcium (500 to 1000 mg/day) starting on the day of infusion. As our study was carried out in a daily practice setting, physicians supplemented patients with cholecalciferol before infusion. The normal median 25OH-vitamin D levels in our study may explain, to some extent, the lack of relationship with APR; our findings are in agreement with those of Chen et al. [26] who supplemented 500 mg calcium and 800 IU vitamin D to all patients. We hypothesise that 25-hydroxyvitamin D supplementation before infusion, when indicated, could prevent reactions. This hypothesis remains to be tested in future trials.

Reid et al. reanalysed the HORIZON-Pivotal Fracture Trial [8, 17], given that the HORIZON study did not assess details about APR. Evaluating racial groups within the HORIZON population, they concluded that race was significantly associated with APR, such that non-Japanese Asians and Pacific Islanders had the highest odds of APR (OR = 3.39) and Latin Americans presented lower odds than other regions (OR = 0.26). In our population, which enrolled Latin Americans only, smoking and diabetes frequencies did not differ between those with and without APR.

One of our main objectives was to examine the association between baseline CTX, ΔCTX, and APR. We hypothesized that the greater the number of blocked osteoclasts, the greater the likelihood that CTX would fall and the higher the likelihood of APR. Bertoldo et al. and Popp et al. [18, 20] investigated the relationship between baseline CTX and acute-phase response, but ΔCTX has never been evaluated before. We found that ΔCTX was significantly greater in patients with APR. One explanation for this finding could be that APR is associated with a higher biochemical response. Therefore, the more significant the interaction, the greater the stimulation, and the higher the inflammatory cytokines production. Subjects previously exposed to oral bisphosphonate potentially had osteoclasts reactivation slightly blocked when subjects received the first zoledronic acid dose. Similarly, the first zoledronic acid use would be associated with a lower APR frequency in subsequent zoledronic acid doses.

Others have described that some inflammatory cytokine levels increase after the first zoledronic acid infusion [9, 11–13]. We found that ΔCTX is associated with APR, and it is known that serum CTX levels are related to osteoclast activity. Consequently, osteoclasts may be the first type of cell to react with bisphosphonates [31]. Additionally, when T lymphocytes are stimulated to produce proinflammatory cytokines before osteoclasts, the latter promotes bone resorption [14]. The interaction of osteoclasts and medications may induce the production of proinflammatory cytokines, which in turn stimulate *γδ* lymphocytes; therefore, the greater the interaction, the greater the stimulation. Hewitt et al. [10] showed that *γδ* lymphocytes could be directly stimulated by bisphosphonates and that statins inhibit such stimuli. However, some *in vivo* studies were unable to replicate these *in vitro* findings [16, 23, 32]. Moreover, previous studies tested monocyte-lineage cells but not osteoclasts or osteal macrophages. Therefore, some questions remain regarding mediation mechanisms and how to mitigate responses that lead to APR.

Because this was a retrospective real-world cohort, we anticipate some limitations. In this type of study, causal inference cannot be established; therefore, conclusions can only be accepted as hypothesis generating, and external validity is generally attainable at the expense of internal validity [27]. The time of exam collection varied, especially for 25-hydroxyvitamin D and CTX, which could have attenuated some effect sizes, although presumably this would have affected both groups and any bias would thus be nondifferential. We did not assess the time patients stopped oral bisphosphonate before their first zoledronic acid infusion, which could interfere with osteoclast blockage. Patients' diabetes profiles were not investigated in this study. The CTX analysis had only 180 patients with full data from a total of 400 patients, limiting power to detect other associations; this could be a potential source of selection bias with respect to the logistic regression analysis. However, sensitivity analysis showed that the exposure was similarly distributed between the groups. Unfortunately, P1NP was unavailable at the study time. Since P1NP is not affected by food intake or glucose status and suffers less by circadian rhythm, it would have been a more accurate tool to corroborate our findings. Additionally, inflammatory cytokine (e.g., IL-1, IL-6, and TNF*α*) would also add insights into the nature of the APR response. However, they were not part of our routine testing to monitor zoledronic acid treatment.

Bone markers and bone mineral mass are independent predictors of fracture. These factors must be combined for fracture evaluation, but they cannot be considered individually. This subject has been studied to define the association between changes in bone turnover markers and bone mineral mass and to analyse whether the former could predict improvement of the latter or predict fracture risk [33–35]. Our next step will be to examine whether acute-phase responders have a higher improvement in bone mineral density and/or fracture risk, since these patients have a higher ΔCTX [36].

## 5. Conclusion

In conclusion, a high incidence of symptoms was observed in response to zoledronic acid infusion. In our study, older age and prior oral bisphosphonate use were associated with a reduced likelihood of APR. Additionally, APR following zoledronic acid infusion was associated with a greater difference in CTX before and after infusion. To our knowledge, this is the first study to describe such findings in a Latin American population with osteoporosis. We suggest that the prevention of reactions would be improved if we could identify potential risk factors and apply appropriate measures. This hypothesis remains to be tested in future research.

## Figures and Tables

**Table 1:** Demographic and clinical characteristics of 428 patients with data on CTX according to the presence or absence of registered APR data about zoledronic acid infusion acute-phase response.

Characteristics	Registered APR data		*p* value
	Yes	No	
Patients, *n* (%)	400	28	
Female, *n* (%)	318 (79.5)	22 (78.6)	0.90
Age (years); mean ± SD	70.3 ± 11.2	72.9 ± 10.9	0.24
Oral bisphosphonate-prior use, *n* (%)	184 (46.0%)	12 (42.9%)	0.75
BMI (kg/m^2^), mean ± SD	25.83 ± 4.8	26.42 ± 5.8	0.73
Diabetes, *n* (%)	59 (14.7%)	08 (28.7%)	0.09
Smoking, *n* (%)			
Never	227 (57.0%)	11 (39.3%)	0.15
Former	141 (35.4%)	13 (46.4%)	
Active	30 (7.5%)	4 (14.3%)	
25-Hydroxyvitamin D, median [25–75^th^ percentile]			
Baseline (ng/mL)	33.6 [25.0, 39.0]	33.9 [25.0, 38.0]	0.83
After infusion (ng/mL)	32.6 [24.0, 38.0]	33.9 [24.8, 41.0]	0.57
Total calcium (mg/dL), median [25–75^th^ percentile]	9.3 [8,9, 9.5]	9.3 [9.1, 9.5]	0.61
PTH, median [25–75^th^ percentile]	55.3 [39.0, 62.0]	52.6 [36.3, 66.0]	0.61
eGFR level (mL/min), mean ± SD	60 ± 16	59 ± 20	0.64
CTX, median [25–75^th^ percentile]			
Baseline, ng/mL	0.517 [0.314, 0.695]	0.617 [0.416, 0.743]	0.13
After infusion, ng/mL	0.218 [0.123, 0.267]	0.351 [0.187, 0.429]	0.06

BMI, body mass index; PTH, parathyroid hormone; eGFR, estimated glomerular filtration rate, measured by CKD-EPI creatinine equation; CTX, carboxy-terminal crosslinked telopeptide of type 1 collagen.

**Table 2:** Demographic and clinical characteristics of 400 patients according to the presence or absence of zoledronic acid infusion acute-phase response.

Characteristics	Acute-phase response		*p* value
	Absent	Present	
Patients, *n* (%)	302 (75.5)	98 (24.5)	
Female, *n* (%)	241 (79.8)	77 (78.6)	0.79
Age (years); mean ± SD	71.4 ± 10.9	67.2 ± 11.6	0.001
Age categories, *n* (%)			
≤66 years	92 (30.5%)	47 (48.0%)	0.001
67–75 years	100 (33.1%)	32 (32.7%)	
>75 years	110 (36.4%)	19 (19.4%)	
Oral bisphosphonate-prior use, *n* (%)	151 (50.0%)	33 (33.7%)	0.005
BMI (kg/m^2^), mean ± SD	25.88 ± 4.88	25.87 ± 4.68	0.99
Diabetes, *n* (%)	47 (15.6%)	12 (12.2%)	0.42
Smoking, *n* (%)			
Never	177 (58.6%)	50 (51.0%)	0.11
Former	98 (32.5%)	43 (43.9%)	
Active	25 (8.3%)	5 (5.1%)	
25-Hydroxyvitamin D, median [25–75^th^ percentile]			
Baseline (ng/mL)	32.0 [25.0, 39.0]	32.0 [26.0, 37.0]	0.91
After infusion (ng/mL)	31.0 [25.0, 39.0]	30.0 [23.0, 36.0]	0.27
Group low-low × normal-normal (*n*)	56 × 44	20 × 7	0.14
Total calcium (mg/dL), median [25–75^th^ percentile]	9.2 [8.9, 9.6]	9.2 [8.9, 9.7]	0.22
PTH, median [25–75^th^ percentile]	49.0 [37.5, 62.0]	52.0 [43.0, 64.0]	0.18
eGFR level (mL/minute), mean ± SD	59 ± 16	62 ± 17	0.17
CTX, median [25–75^th^ percentile]			
Baseline, ng/mL	0.430 [0.249, 0.700]	0.535 [0.375, 0.679]	0.026
After infusion, ng/mL	0.182 [0.125, 0.267]	0.170 [0.122, 0.274]	0.59
ΔCTX median [25–75^th^ percentile]	−53.7 [−72.1, −23.4]	−69.3 [−76.2, −49.7]	0.002

BMI, body mass index; PTH, parathyroid hormone; eGFR, estimated glomerular filtration rate, measured by CKD-EPI creatinine equation; CTX, carboxy-terminal crosslinked telopeptide of type 1 collagen; ΔCTX: delta carboxy-terminal crosslinked telopeptide of type 1 collagen.

**Table 3:** Associations of clinical and demographic variables with acute-phase response following zoledronic acid infusion.

Characteristics	Unadjusted		Model 1^*∗*^		Model 2^*∗∗*^	
	OR (95% CI)	*p* value	OR (95% CI)	*p* value	OR (95% CI)	*p* value
Age	0.97 (0.95–0.99)	0.002	0.94 (0.92–0.97)	<0.001	0.95 (0.92–0.98)	0.004
Prior oral BP	0.51 (0.31–0.82)	0.005	0.33 (0.16–0.65)	0.002	0.28 (0.12–0.64)	0.003
CTX	1.61 (1.04–2.48)	0.031	1.20 (0.72–1.98)	0.473	—	—
ΔCTX	0.53 (0.36–0.79)	0.002	—	—	0.60 (0.39–0.92)	0.019

BP, bisphosphonate; CTX (baseline), carboxy-terminal crosslinked telopeptide of type 1 collagen; ΔCTX: delta carboxy-terminal crosslinked telopeptide of type 1 collagen, calculated as log; OR: odds ratio. ^*∗*^ Model 1 included log-transformed baseline CTX, age, and prior oral bisphosphonate use. This study included 258 patients with complete data. ^*∗∗*^ Model 2 included log-transformed ΔCTX, age, and prior use of oral bisphosphonate. This study included 180 patients with complete data.

**Table 4:** Demographic and clinical characteristics of 180 patients with data on CTX according to the presence or absence of acute-phase response following zoledronic acid infusion.

Characteristics	Acute-phase response		*p* value
	Absent	Present	
Patients, *n* (%)	132 (73.3)	48 (26.7)	
Female, *n* (%)	107 (81.1)	40 (83.3)	0.73
Age (years); mean ± SD	71.58 ± 10.67	66.98 ± 12.77	0.016
Age categories, *n* (%)			
≤66 years	40 (30.3%)	22 (45.8%)	0.035
67–75 years	45 (34.1%)	18 (37.5%)	
>75 years	47 (35.6%)	8 (16.7%)	
Oral bisphosphonate-prior use, *n* (%)	69 (52.3%)	13 (27.1%)	0.003
BMI (kg/m^2^), mean ± SD	26.27 ± 4.61	26.14 ± 4.62	0.87
Diabetes, *n* (%)	26 (19.7%)	7 (14.6%)	0.43
Smoking, *n* (%)			
Never	76 (57.6%)	24 (50.0%)	0.73
Former	7 (5.3%)	3 (6.2%)	
Active	48 (36.4%)	21 (43.8%)	
25-Hydroxyvitamin D, median [25–75^th^ percentile]			
Baseline (ng/mL)	31.0 [25.0, 37.0]	31.0 [26.0, 37.0]	0.61
After infusion (ng/mL)	30.0 [24.0, 40.0]	30.0 [23.0, 37.0]	0.42
Group low-low × normal-normal (*n*)	33 × 49	17 × 13	0.93
Total calcium (mg/dL), median [25–75^th^ percentile]	9.2 [8.9, 9.6]	9.4 [9.1, 9.7]	0.15
PTH, median [25–75^th^ percentile]	51.0 [41.0, 63.0]	52.0 [42.0, 67.0]	0.18
eGFR level (mL/minute), mean ± SD	57.68 ± 14.80	63.09 ± 19.76	0.06
CTX, median [25–75^th^ percentile]			
Baseline, ng/mL	0.435 [0.251, 0.690]	0.531 [0.380, 0.699]	0.024
After infusion, ng/mL	0.182 [0.130, 0.264]	0.156 [0.122, 0.251]	0.26
ΔCTX median [25–75^th^ percentile]	−53.7 [−72.1, −23.4]	−69.3 [−76.2, −49.7]	0.002

BMI, body mass index; PTH, parathyroid hormone; eGFR, estimated glomerular filtration rate, measured by CKD-EPI creatinine equation; CTX, carboxy-terminal crosslinked telopeptide of type 1 collagen; ΔCTX: delta carboxy-terminal crosslinked telopeptide of type 1 collagen.

## Data Availability

Our data are not freely available due to our institution's policy.

## Supplementary Materials

Supplementary Materials The supplementary material represents the analysis of women and men subgroups.
