# Supplementary material for: Characteristics Associated with Acute-Phase Response following First Zoledronic Acid Infusion in Brazilian Population with Osteoporosis
Source: J Osteoporos. 2021 Dec 29;2021:9492883. doi: 10.1155/2021/9492883 (PMC8731262; doi:10.1155/2021/9492883)
Supplement: Supplementary Materials — The supplementary material represents the analysis of women and men subgroups. [file 9492883.f1.docx]

**SUPPLEMENTAL MATERIAL**

Supplemental Table 1. Demographic and clinical characteristics of 318 women according to the presence or absence of zoledronic acid infusion acute-phase response.

| Characteristics | Acute-phase response | | |
| --- | --- | --- | --- |
|  | Absent | Present | p-value |
| Patients, n (%) | 241 (76) | 77 (24) |  |
| Age (years); mean ± SD | 71.7 ± 10.5 | 67.1 ± 10.7 | <0.001 |
| Oral bisphosphonate - prior use, n (%) | 132 (55%) | 30 (39%) | 0.016 |
| BMI (kg/m^2^), mean ± SD | 26.4 ± 4.9 | 26.3 ± 4.5 | 0.93 |
| Diabetes, n (%) | 37 (15%) | 9 (12%) | 0.43 |
| Smoking, n (%) |  |  | 0.02 |
| Never | 150 (63%) | 41 (53%) |  |
| Former | 20 (8.4%) | 2 (2.6%) |  |
| Active | 69 (29.9%) | 34 (44%) |  |
| 25-hidroxyvitamin D, median [25-75^th^ percentile] |  |  |  |
| Baseline (ng/mL) | 31.0 [25.0, 38.0] | 30.0 [26.0, 37.0] | 0.86 |
| After infusion (ng/mL) | 30.0 [24.0, 38.0] | 30.0 [23.0, 36.0] | 0.28 |
| Total calcium (mg/dL), median [25-75^th^ percentile] | 9.2 [8.9, 9.6] | 9.3 [8.9, 9.7] | 0.52 |
| PTH, median [25-75^th^ percentile] | 51.0 [40.0, 64.0] | 53.0 [44.0, 67.0] | 0.43 |
| eGFR level (mL/minute), mean±SD | 57 ± 15 | 60 ± 15 | 0.17 |
| CTX, median [25-75^th^ percentile] |  |  |  |
| Baseline, ng/mL | 0.43  [0.21, 0.70] | 0.53  [0.35, 0.70] | 0.10 |
| After infusion, ng/mL | 0.20  [0.12, 0.27] | 0.21  [0.12, 0.29] | 0.61 |
| ΔCTX median [25-75^th^ percentile] | -50.0  [-71.0, -21.9] | -66.3  [-74.8, -46.3] | 0.016 |

Abbreviations: BMI, body mass index; PTH, parathyroid hormone; eGFR, estimated glomerular filtration rate, measured by CKD-EPI creatinine equation; CTX, carboxy-terminal crosslinked telopeptide of type 1 collagen; ΔCTX: delta carboxy-terminal crosslinked telopeptide of type 1 collagen.

Supplemental Table 2. Demographic and clinical characteristics of 318 men according to the presence or absence of zoledronic acid infusion acute-phase response.

| Characteristics | Acute-phase response | | |
| --- | --- | --- | --- |
|  | Absent | Present | p-value |
| Patients, n (%) | 61 (74) | 21 (26) |  |
| Age (years); mean ± SD | 70.1 ± 12.3 | 67.4 ± 14.9 | 0.42 |
| Oral bisphosphonate - prior use, n (%) | 19 (31%) | 3 (14%) | 0.13 |
| BMI (kg/m^2^), mean ± SD | 23.8 ± 4.2 | 24.2 ± 5.0 | 0.73 |
| Diabetes, n (%) | 10 (16%) | 3 (14%) | 0.82 |
| Smoking, n (%) |  |  | 0.71 |
| Never | 27 (44%) | 9 (43%) |  |
| Former | 5 (8%) | 3 (14%) |  |
| Active | 29 (48%) | 9 (43%) |  |
| 25-hidroxyvitamin D, median [25-75^th^ percentile] |  |  |  |
| Baseline (ng/mL) | 34.0 [25.0, 42.5] | 35.0 [30.0, 40.0] | 0.82 |
| After infusion (ng/mL) | 32.0 [25.0, 41.0] | 32.0 [26.0, 38.0] | 0.83 |
| Total calcium (mg/dL), median [25-75^th^ percentile] | 9.1 [8.8, 9.5] | 9.1 [9.0, 9.7] | 0.12 |
| PTH, median [25-75^th^ percentile] | 42.0 [31.0, 55.0] | 50.5 [40.0, 57.5] | 0.08 |
| eGFR level (mL/minute), mean±SD | 68 ± 18 | 69 ± 23 | 0.91 |
| CTX, median [25-75^th^ percentile] |  |  |  |
| Baseline, ng/mL | 0.52  [0.31, 0.79] | 0.59  [0.51, 0.70] | 0.21 |
| After infusion, ng/mL | 0.20  [0.11, 0.28] | 0.21  [0.12, 0.31] | 0.86 |
| ΔCTX median [25-75^th^ percentile] | -65.4  [-73.9, -45.0] | -75.3  [-82.8, -73.1] | 0.007 |

Abbreviations: BMI, body mass index; PTH, parathyroid hormone; eGFR, estimated glomerular filtration rate, measured by CKD-EPI creatinine equation; CTX, carboxy-terminal crosslinked telopeptide of type 1 collagen; ΔCTX: delta carboxy-terminal crosslinked telopeptide of type 1 collagen.

Supplemental Table 3. Associations of clinical and demographic variables with acute phase response following zoledronic acid infusion.

| Characteristics | Unadjusted | | Model 1* | | Model 2** | |
| --- | --- | --- | --- | --- | --- | --- |
|  | OR  (95% CI) | p-value | OR  (95% CI) | p-value | OR  (95% CI) | p-value |
| Age | 0.97  (0.95-0.99) | 0.002 | 0.94  (0.92-0.97) | <0.001 | 0.95  (0.92-0.98) | 0.004 |
| Prior oral BP | 0.51  (0.31-0.82) | 0.005 | 0.33  (0.16-0.65) | 0.002 | 0.28  (0.12-0.64) | 0.003 |
| CTX | 1.61  (1.04-2.48) | 0.031 | 1.20  (0.72-1.98) | 0.473 | -- | -- |
| ΔCTX | 0.53  (0.36-0.79) | 0.002 | -- | -- | 0.60  (0.39-0.92) | 0.019 |

Abbreviations: BP, bisphosphonate; CTX (baseline), carboxy-terminal crosslinked telopeptide of type 1 collagen; ΔCTX: delta carboxy-terminal crosslinked telopeptide of type 1 collagen, calculated as log; OR: odds ratio

* Model 1 included log-transformed baseline CTX, age, sex, and prior oral bisphosphonate use. This study included 258 patients with complete data.

** Model 2 included log transformed ΔCTX, age, sex and prior use of oral bisphosphonate. This study included 180 patients with complete data.
